# Ambient Temperature and Prevalence of Obesity: A Nationwide Population-Based Study in Korea

**DOI:** 10.1371/journal.pone.0141724

**Published:** 2015-11-02

**Authors:** Hae Kyung Yang, Kyungdo Han, Jae-Hyoung Cho, Kun-Ho Yoon, Bong-Yun Cha, Seung-Hwan Lee

**Affiliations:** 1 Division of Endocrinology and Metabolism, Department of Internal Medicine, Seoul St. Mary’s Hospital, College of Medicine, The Catholic University of Korea, Seoul, Korea; 2 Department of Medical Statistics, College of Medicine, The Catholic University of Korea, Seoul, Korea; McMaster University, CANADA

## Abstract

**Background:**

Recent studies have suggested a possible association between outdoor or indoor temperature and obesity. We aimed to examine whether ambient temperature is associated with the prevalence of obesity or abdominal obesity in the Korean population.

**Methods:**

Data on anthropometric, socio-demographic, laboratory and lifestyle factors were retrieved from National Health Insurance System data obtained in 2009–2010. Thirty years (1981 to 2010) of meteorological parameters for 71 observation areas were acquired from the Korea Meteorological Administration. Included in this analysis were 124,354 individuals. A body mass index (BMI) ≥ 25 kg/m^2^ and a waist circumference (WC) ≥ 90 cm (men) or 85 cm (women) were considered to represent obesity and abdominal obesity, respectively.

**Results:**

The mean annual temperature (MAT) ranged from 6.6°C to 16.6°C, and BMI was positively correlated with MAT (r = 0.0078, *P* = 0.0065). WC was positively correlated with MAT (r = 0.0165, *P* < 0.0001) and negatively correlated with the number of days with mean temperature < 0°C (DMT0; r = –0.0129, *P* = 0.0002). After adjusting for age, sex, smoking status, alcohol consumption, exercise, income, residential area and altitude, the odds ratios (95% CI) for obesity and abdominal obesity in the highest quintile MAT group were 1.045 (1.010, 1.081) and 1.082 (1.042, 1.124), respectively, compared with the lower four quintiles of the MAT group. Similarly, subjects in the area of the lowest quintile of DMT0 had significantly higher odds of abdominal obesity compared with the higher four quintile groups of DMT0.

**Conclusion:**

This study finds an association between ambient temperature and prevalence of obesity in the Korean population when controlling for several confounding factors. Adaptive thermogenesis might be a possible explanation for this phenomenon.

## Introduction

Excess body weight is an important risk factor for mortality and morbidity causing nearly 3 million deaths every year worldwide [1]. Compared to 1980, the worldwide trends show heightened levels of mean body mass index (BMI) by 0.4 kg/m^2^/decade for men and 0.5 kg/m^2^/decade for women [2], and the epidemic of obesity and associated comorbidities became a major global public health challenge [3–5]. Higher energy intake and lower energy expenditure owing to unhealthy diets and sedentary lifestyle are major contributors to the increasing prevalence of obesity. However, other conditions such as genetic, biological and environmental factors may also contribute to this trend [6]. For instance, several investigators have proposed a hypothesis that an increase in indoor temperatures may be one of the factors that has contributed to the current obesity epidemic by negatively affecting energy expenditure [7, 8].

Most people in the developed world live and work in a relatively narrow temperature range of comfortable ambient temperatures known as the thermoneutral zone, which is approximately 23.0°C for humans [9, 10]. Little or no energy is required to stabilize the body’s core temperature in a thermoneutral zone. However, with a decrease in ambient temperature below the thermoneutral zone, an extra energy expenditure of 105 to 156 kJ/day/1°C is needed to maintain thermal homeostasis [11]. Previously, only a few studies have suggested a possible association between outdoor or indoor temperature and obesity [12–16], and most of the studies are from Western populations.

In this study, we aimed to examine whether ambient temperature is associated with the prevalence of obesity or abdominal obesity using nationally representative data of the Korean population.

## Subjects and Methods

### Data source and study population

The National Health Insurance System (NHIS) contains comprehensive health-related information for approximately 50 million Koreans and is composed of an eligibility database (age, sex, socioeconomic variables, area of residency, type of eligibility, income level, etc.), a medical treatment database (based on the medical bills that were claimed by medical service providers for their medical expense claims), a health examination database (results of general health examinations and questionnaires on lifestyle and behavior) and a medical care institution database (types of medical care institutions, province location, equipment and number of physicians). The source population for this system is the Health Insurance Review and Assessment (HIRA) service. Under the NHIS, Koreans are entitled to medical coverage as either an employee or a member of a community [17, 18]. Healthcare providers are required to submit reports on medical services provided under the health insurance policies to the HIRA service for a review of the medical costs incurred. Accordingly, the HIRA database contains information on all insurance claims for approximately 97.0% of the population in Korea.

A customized database from NHIS that includes approximately 2.2% subjects of the Korean population was used in this study. Subjects were selected by stratified random sampling to represent the total population. Of the 235,148 participants who underwent health examinations in the year 2009 and 2010, those with missing variables (n = 55,067) and those living in the area of missing meteorological data (n = 55,727) were excluded. Ultimately, the study population consisted of 124,354 subjects. This study was approved by the Institutional Review Board of The Catholic University of Korea (No. KC15EISI0431). Anonymized and de-identified information was used for analyses, and therefore informed consent was not obtained.

### Meteorological parameters

**Fig 1:**
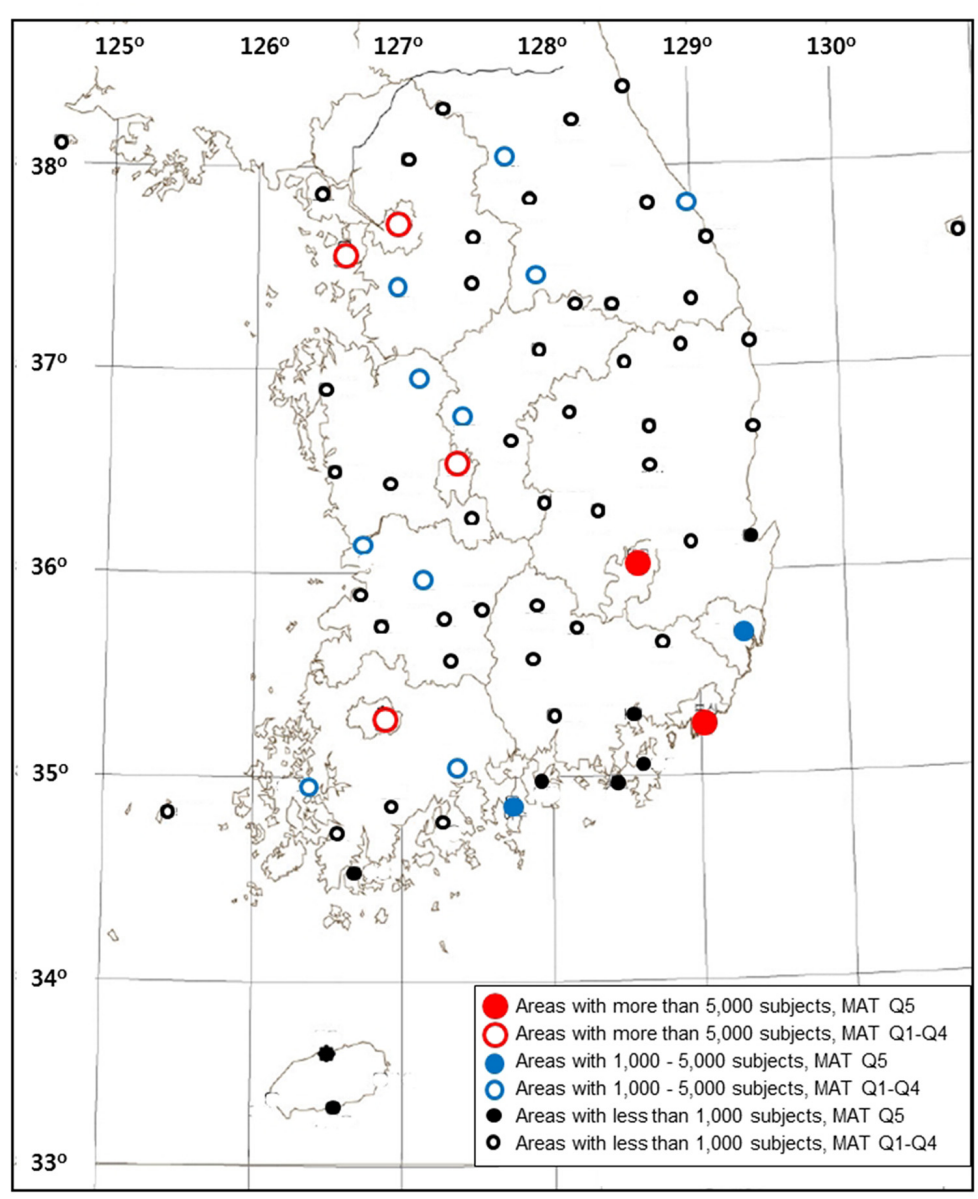
Map of the Republic of Korea showing the 71 observation areas included in the study, grouped according to their number of subjects participated and mean annual temperature (MAT) quintiles.

The data on mean annual temperature (MAT), the number of days with mean temperature (DMT) < –5°C (DMT–5), < 0°C (DMT0), ≥ 5°C (DMT5) and ≥ 25°C (DMT25), altitude (height of observation area above mean sea level) and information on the observation area were extracted from the Climatological Normals of Korea published by the Korea Meteorological Administration in 2011. These data were obtained for 73 observation stations that are located throughout South Korea and have latitudes ranging from 33° 14’ to 38° 15’. The statistics are based on a 30-year period (1981 to 2010). In this study, data from 71 observation areas were merged with the NHIS data (two observation areas were excluded because of a missing corresponding health examination database) (Fig 1).

### Measurements

Anthropometric measurements were performed while the subjects were wearing light clothing. BMI was calculated as the subject’s weight in kilograms divided by the square of the subject’s height in meters. Obesity and normal weight were defined as BMI ≥ 25 and < 25 kg/m^2^, respectively [19]. Waist circumference was measured midway between the lower rib margin and the iliac crest in a standing position. The cutoff value of waist circumference for abdominal obesity was ≥ 90 cm for men and ≥ 85 cm for women [20, 21]. Blood pressure (BP) was measured while the individual was in a seated position after at least a 5-min period of rest. Smoking status was categorized as non-smoker, ex-smoker or current smoker. Alcohol drinking status was categorized as non-drinker or drinker based on the questionnaire asking the frequency of alcohol consumption per week. Those who were drinking once a week or more frequently were considered as alcohol drinkers. Regular exercise was defined as strenuous physical activity that was performed for at least 20 min three times a week. Income level was dichotomized at the lower 20%. Blood samples were drawn after an overnight fasting and measured for serum levels of glucose, total cholesterol, triglycerides, high-density lipoprotein (HDL)-cholesterol, hemoglobin, serum creatinine, aspartate aminotransferase (AST), alanine aminotransferase (ALT) and gamma-glutamyl transpeptidase (γ-GTP). Low-density lipoprotein (LDL)-cholesterol was calculated using the Friedewald formula [22]. Hospitals performing health examinations were certified by the NHIS and subjected to regular quality control.

### Statistical analyses

The data are expressed as the means ± standard deviations (SDs), median (25th–75th percentiles) or as percentages. The characteristics of the study subjects according to MAT quintile groups (lower four quintiles vs highest quintile) were compared using *t*-tests, the Mann-Whitney test or chi-squared tests. The cutoff value of the highest quintiles of MAT was 14.1°C. The correlation between BMI, waist circumference and meteorological parameters were tested using regression analysis, and the Spearman correlation coefficient was calculated. The mean values of meteorological parameters in the 71 observation areas were separately plotted, and the correlations with BMI and waist circumference were analyzed accounting for sampling weights of each area. The risks of obesity and abdominal obesity according to the MAT quintile groups were tested using the lower four quintile MAT groups as a reference group. To account for possible confounding factors, age and sex were adjusted in Model 1 and alcohol consumption, smoking, exercise, income, residential area and altitude were further adjusted in Model 2. The risks of obesity and abdominal obesity according to the DMT0 quintile groups were tested using the highest four quintile DMT0 groups as reference groups after adjusting for the abovementioned variables. A *P* value < 0.05 was considered significant. The statistical analyses were performed using SAS version 9.3 (SAS Institute Inc., Cary, NC, USA).

## Results

### Baseline characteristics according to MAT quintile groups

The MAT ranged from 6.6°C to 16.6°C. The study subjects were categorized according to the MAT quintile groups (Table 1). Compared to subjects in the lower four quintile groups, subjects in the highest quintile of MAT (≥ 14.1°C) demonstrated higher BMI, waist circumference, diastolic blood pressure, fasting blood glucose, triglycerides, LDL-cholesterol, hemoglobin and γ-GTP levels and lower HDL-cholesterol levels. These differences were slight but significant. Age distribution, sex, smoking, alcohol consumption, exercise and income status also differed between subjects in the highest quintile of MAT vs subjects in the lower four quintile groups.

**Table 1:** Characteristics of study subjects according to mean ambient temperature quintile groups.

	MAT Q1–-Q4 (< 14.1℃)	MAT Q5 (≥ 14.1℃)	*P*
Number	106,476	17,878	
Age (years, %)			< 0.001
20–39	32.0	31.0	
40–59	48.0	47.3	
≥ 60	20.0	21.7	
Sex (men, %)	50.4	49.2	0.005
Height (cm)	163.8 ± 9.3	164.2 ± 9.3	< 0.001
Weight (kg)	63.8 ± 11.7	64.4 ± 11.5	< 0.001
Body mass index (kg/m^2^)	23.7 ± 3.2	23.8 ± 3.2	< 0.001
Waist circumference (cm)	80.1 ± 9.2	80.7 ± 8.9	< 0.001
Systolic BP (mmHg)	122.4 ± 15.1	122.5 ± 14.9	0.495
Diastolic BP (mmHg)	76.3 ± 10.1	76.5 ± 10.0	0.015
Fasting glucose (mg/dL)	97.4 ± 24.2	98.0 ± 23.6	0.001
Total cholesterol (mg/dL)	195.3 ± 36.9	195.8 ± 36.5	0.079
Triglycerides (mg/dL)	109 (74–162)	111 (76–166)	< 0.001
HDL–cholesterol (mg/dL)	56.5 ± 29.5	55.1 ± 26.4	< 0.001
LDL–cholesterol (mg/dL)	113.5 ± 38.0	115.0 ± 39.5	< 0.001
Hemoglobin (g/dL)	13.9 ± 1.6	14.1 ± 1.6	< 0.001
Serum creatinine (mg/dL)	1.10 ± 1.34	1.16 ± 1.50	< 0.001
AST (U/L)	22 (19–28)	23 (19–28)	0.690
ALT (U/L)	20 (15–29)	20 (15–29)	0.0715
γ-GTP (U/L)	23 (16–39)	24 (16–41)	< 0.001
Smoking (%)			< 0.001
None	60.0	59.3	
Ex-smoker	14.0	15.5	
Current smoker	26.0	25.2	
Alcohol drinking (yes, %)	48.8	50.2	< 0001
Regular exercise (%)	15.1	15.8	0.002
Income (low)	22.5	25.8	< 0.001

Data are expressed as the means ± SD, % or median (25th-75th percentiles). AST = aspartate aminotransferase; ALT = alanine aminotransferase; BP = blood pressure; γ-GTP = gamma glutamyltransferase; HDL = high-density lipoprotein; LDL = low-density lipoprotein; MAT = mean annual temperature.

### Correlation between BMI, waist circumference and meteorological parameters

The BMI of enrolled participants was positively correlated with MAT (r = 0.0078, *P* = 0.0065) and DMT5 (r = 0.0070, *P* = 0.0142). Waist circumference was positively correlated with MAT (r = 0.0165, *P* < 0.0001) and DMT5 (r = 0.0157, *P* < 0.0001) and negatively correlated with DMT–5 (r = –0.0123, *P* = 0.0002) and DMT0 (r = –0.0129, *P* = 0.0002). However, no significant correlation was observed between BMI or waist circumference and altitude (Table 2).

**Table 2:** The correlation between body mass index, waist circumference and meteorological parameters.

	Body mass index		Waist circumference	
	r	*P*	r	*P*
MAT	0.0078	0.0065	0.0165	< 0.0001
DMT–5	–0.0047	0.1010	–0.0123	0.0002
DMT0	–0.0040	0.1579	–0.0129	0.0002
DMT5	0.0070	0.0142	0.0157	< 0.0001
DMT25	–0.0024	0.3998	–0.0058	0.0992
Altitude	0.0042	0.1357	0.0005	0.4742

MAT = mean annual temperature; DMT–5 = number of days with mean temperature < –5°C; DMT0 = number of days with mean temperature < 0°C; DMT5 = number of days with mean temperature ≥ 5°C; DMT25 = number of days with mean temperature ≥ 25°C; Altitude = height of observation field above mean sea level.

**Fig 2:**
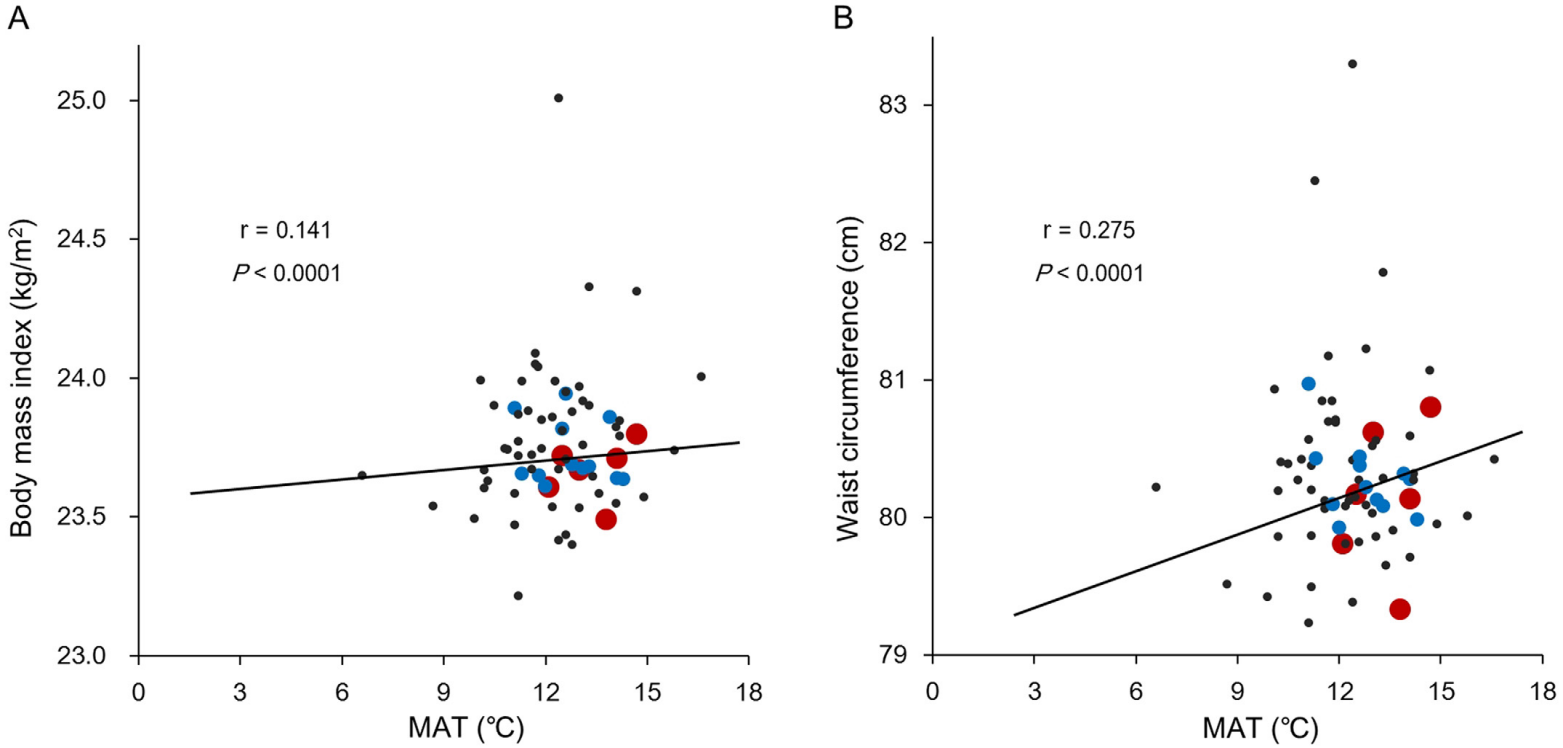
Correlation between body mass index (A) and waist circumference (B) with mean annual temperature (MAT) based on 71 observation areas. Red dot: areas with more than 5,000 subjects; blue dot: areas with 1,000–5,000 subjects; black dot: areas with less than 1,000 subjects.

**Fig 3:**
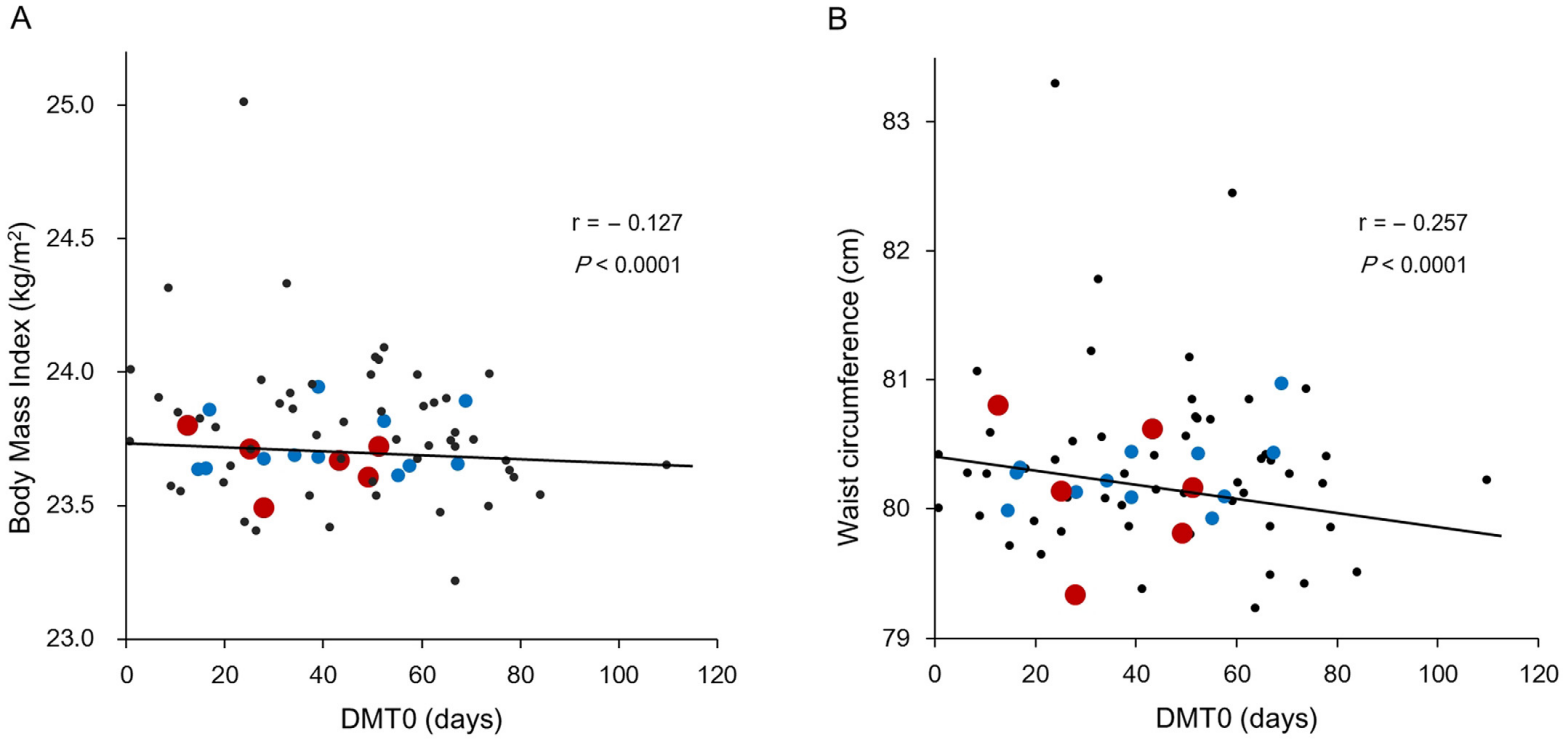
Correlation between body mass index (A) and waist circumference (B) with the number of days with mean temperature < 0°C (DMT0) based on 71 observation areas. Red dot: areas with more than 5,000 subjects; blue dot: areas with 1,000–5,000 subjects; black dot: areas with less than 1,000 subjects.

Next, the values of the MAT and DMT0 for each of the 71 observation areas were plotted against their mean BMI and waist circumference values. After accounting for sampling weights of each area, both the BMI and waist circumference positively correlated with MAT and exhibited correlation coefficients of 0.141 (*P* < 0.0001) and 0.275 (*P* < 0.0001), respectively (Fig 2). BMI (r = –0.127, *P* < 0.0001) and waist circumference (r = –0.257, *P* < 0.0001) were negatively correlated with DMT0 (Fig 3).

### The risk of obesity and abdominal obesity according to MAT and DMT0 quintile groups

Compared to subjects in the lower four quintiles of the MAT, those in the highest quintile group exhibited a 1.047 times higher odds of obesity after adjusting for age and sex in Model 1. Further adjustment for alcohol consumption, smoking, exercise, income, residential area and altitude (Model 2) did not attenuate this association and demonstrated an odds ratio (95% CI) of 1.045 (1.010, 1.081) for obesity. Similarly, the odds ratios (95% CI) for abdominal obesity were 1.080 (1.041, 1.122) and 1.082 (1.042, 1.124) in Models 1 and 2, respectively (Table 3).

**Table 3:** The risk of obesity and abdominal obesity according to MAT quintile groups.

Variables	Obesity		Abdominal obesity	
	OR (95% CI)	*P*	OR (95% CI)	*P*
Model 1				
MAT (Q5 vs Q1–Q4)	1.047 (1.012, 1.083)	0.0073	1.080 (1.041, 1.122)	< 0.0001
Age (per 5 year increment)	1.001 (0.997, 1.005)	0.6399	0.999 (0.995, 1.003)	0.5264
Sex (female)	1.000 (0.976, 1.024)	0.9982	2.607 (2.536, 2.681)	< 0.0001
Model 2				
MAT (Q5 vs Q1–Q4)	1.045 (1.010, 1.081)	0.0114	1.082 (1.042, 1.124)	< 0.0001
Age (per 5 year increment)	1.001 (0.997, 1.005)	0.7028	0.999 (0.994, 1.003)	0.5160
Sex (female) Smoking	1.001 (0.977, 1.025)	0.9286	2.673 (2.599, 2,749)	< 0.0001
Ex-smoker vs non-smoker	1.565 (1.510, 1.621)	< 0.0001	2.547 (2.450, 2.648)	< 0.0001
Current smoker vs non-smoker	1.312 (1.273, 1.353)	< 0.0001	1.993 (1.926, 2.061)	< 0.0001
Alcohol drinking	1.014 (0.988, 1.041)	0.2867	1.140 (1.107, 1.175)	< 0.0001
Exercise	1.170 (1.132, 1.209)	< 0.0001	1.091 (1.051, 1.132)	< 0.0001
Income (higher 80% vs lower 20%)	0.992 (0.964, 1.020)	0.5623	0.998 (0.966, 1.031)	0.9069
Residential area (rural vs. metropolitan city)	1.003 (0.976, 1.031)	0.8477	0.986 (0.956, 1.017)	0.3786
Altitude (per 10 m increment)	0.998 (0.987, 1.010)	0.7750	1.005 (0.992, 1.018)	0.4675

MAT = mean annual temperature; Cutoff value of the highest quintile of mean ambient temperature is 14.1℃. Obesity is defined as BMI ≥ 25 kg/m^2^. Abdominal obesity is defined as WC ≥ 90 cm for men and ≥ 85 cm for women. Model 1: Adjusted for age and sex. Model 2: Adjusted for Model 1 + alcohol drinking, smoking, exercise, income, residential area and altitude.

Compared to subjects in the higher four quintiles of DMT0, those in the lowest quintile group demonstrated a 1.062 times higher odds of abdominal obesity after adjusting for age and sex. Further adjustment for alcohol consumption, smoking, exercise, income, residential area and altitude demonstrated an odds ratio (95% CI) of 1.063 (1.027, 1.100). However, subjects in the lowest quintile of DMT0 did not exhibit a significantly higher odds for obesity compared to those in the higher four quintile groups (Table 4).

**Table 4:** The risk of obesity and abdominal obesity according to DMT0 quintile groups.

Variables	Obesity		Abdominal obesity	
	OR (95% CI)	*P*	OR (95% CI)	*P*
Model 1				
DMT0 (Q1 vs Q2–Q5)	1.030 (0.999, 1.062)	0.0553	1.062 (1.027, 1.098)	0.0005
Age (per 5 year increment)	1.001 (0.997, 1.005)	0.6464	0.999 (0.994, 1.003)	0.5085
Sex (female)	1.000 (0.977, 1.024)	0.9917	2.608 (2.536, 2.681)	< 0.0001
Model 2				
DMT0 (Q1 vs Q2–Q5)	1.027 (0.996, 1.059)	0.0915	1.063 (1.027, 1.100)	0.0005
Age (per 5 year increment)	1.001 (0.997, 1.005)	0.7043	0.999 (0.994, 1.003)	0.5020
Sex (female) Smoking	1.001 (0.977, 1.025)	0.9238	2.673 (2.599, 2,749)	< 0.0001
Ex-smoker vs non-smoker	1.565 (1.510, 1.622)	< 0.0001	2.548 (2.451, 2.648)	< 0.0001
Current smoker vs non-smoker	1.312 (1.273, 1.353)	< 0.0001	1.992 (1.926, 2.061)	< 0.0001
Alcohol drinking	1.014 (0.988, 1.041)	0.2827	1.140 (1.107, 1.175)	< 0.0001
Exercise	1.170 (1.133, 1.209)	< 0.0001	1.091 (1.051, 1.132)	< 0.0001
Income (higher 80% vs lower 20%)	0.991 (0.963, 1.020)	0.5381	0.998 (0.966, 1.030)	0.8819
Residential area (rural vs. metropolitan city)	1.000 (0.973, 1.028)	0.9788	0.982 (0.952, 1.013)	0.2595
Altitude (per 10 m increment)	0.999 (0.987, 1.010)	0.8289	1.006 (0.993, 1.019)	0.3416

DMT0 = number of days with mean temperature < 0°C; Cutoff value of the lowest quintile of DMT0 is 25 days. Obesity is defined as BMI ≥ 25 kg/m^2^. Abdominal obesity is defined as WC ≥ 90 cm for men and ≥ 85 cm for women. Model 1: Adjusted for age and sex. Model 2: Adjusted for Model 1 + alcohol drinking, smoking, exercise, income, residential area and altitude.

## Discussion

Using nationally representative data of the Korean population, we demonstrated significant associations between obesity or abdominal obesity and ambient temperature. BMI and waist circumference were positively correlated with MAT and negatively correlated with DMT0. The odds for obesity and abdominal obesity were 1.045 and 1.082 times higher in subjects living in the highest quintile MAT group compared to those living in the lower four quintile MAT groups. Similarly, subjects in the area of the lowest quintile of DMT0 had significantly higher odds of abdominal obesity compared with the higher four quintile groups of DMT0. These results suggest that high ambient temperature might be associated with an approximate 5% increased odds of obesity in the Korean population.

Previous studies regarding an association between ambient temperature and BMI are scarce. In Tibetans, BMI, waist circumference and waist-to-height ratio were lower among subjects living at higher altitude compared to those living at lower altitude, and this finding was explained by a possible catabolic effect of low temperature and low oxygen in higher altitude [12]. Valdés et al. reported a positive association between ambient temperature and obesity using a national, cross-sectional population-based survey in Spain [13]. Voss et al. demonstrated a parabolic relationship between mean annual temperature and obesity using nationally representative data specific to US adults [14]. In this study, a maximum prevalence for obesity was observed in counties with average temperatures near 18°C, while the extremes of temperature category tended to the lowest odds for obesity. In another study, BMI levels of those residing in indoor temperatures above 23°C were lower than those living in temperatures below 19°C, suggesting an inverse correlation between BMI and ambient temperature using data from a nationally representative health survey administered in England [15]. In addition to these cross-sectional studies, one longitudinal observation study was conducted among Dutch children. However, the data from the Dutch Prevention and Incidence of Asthma and Mite Allergy (PIAMA) birth cohort did not support any significant associations between indoor temperature and BMI z-scores in children between the ages of 3 months and 11 years [16].

The discrepancy surrounding ambient temperature and obesity might be a consequence of the different ranges in mean temperatures, different altitudes of observed areas, different ethnic groups, and the heterogeneous adjustment for confounding variables in previous studies. However, although some reports do not include a significant number of locations with extreme ambient temperatures [13, 15], they generally support the concept of parabolic association between ambient temperature and obesity [14], with the highest prevalence of obesity around 18–20°C. In our study, the range of mean annual ambient temperatures was between 6.6°C and 16.6°C, and therefore, extremely high or low temperatures were not included. Consistent with a previous report by Valdés et al. wherein the range of ambient temperatures was between 10.4°C and 21.3°C [13], our results once again demonstrate a positive correlation between ambient temperature and the prevalence of obesity. In our study, compared to the lowest four MAT quintile groups (6.6 to 14.1°C), the OR for obesity was 1.045 for the highest MAT quintile group (14.1 to 16.6°C), while Valdés et al., in a study conducted among the Spanish population, reported 1.20, 1.35 and 1.38 times greater odds for obesity in quartiles 2 (14.5 to 15.5°C), 3 (15.5 to 17.8°C) and 4 (17.8 to 21.3°C), respectively, compared to the lowest quartile (10.4 to 14.5°C). Considering several differences, including the prevalence and the diagnostic criteria of obesity and the ranges in temperatures, these two studies suggest that the association between ambient temperature and obesity is meaningful in a relatively wide range of populations living in different environments and having different characteristics.

In contrast to previous studies that included high elevation areas [12, 14], our data do not indicate a significant correlation between the prevalence of obesity and altitude. This could be explained by the lower range of altitudes in our observation areas (2.3 to 772.6 m). Indeed, the odds for obesity in a US study did not differ between mildly elevated areas (0.50 to 0.99 km) and areas located at lower altitudes [14].

Several lines of evidence have suggested a close link between ambient temperature and obesity or energy expenditure in humans. In the range of 15 to 28°C which encompasses normal indoor temperature, energy expenditure was negatively associated with the thermal environment [7]. In addition, a rough calculation using a theoretical thermodynamic model indicated an additional caloric expenditure that corresponds to 5.1 to 7.3 kg of fat burning per year when the temperature is lowered by 5°C [10]. Cold exposure is the natural afferent signal for brown adipose tissue, and several reports have demonstrated an association between brown adipose tissue activity and favorable metabolic profiles [23–26]. In contrast to obligatory thermogenesis, facultative thermogenesis is activated in response to cold or diet and is composed of cold-induced shivering or nonshivering thermogenesis, voluntary or non-exercise activity thermogenesis and some part of diet-induced thermogenesis [27]. Among them, cold-induced thermogenesis accounts for an approximate 10% increase in energy expenditure, which is related to the activity of brown adipose tissue or the browning of white adipose tissue [28–31]. In rodents, brown adipose tissue is known to function during cold-induced, non-shivering thermogenesis and facultative diet-induced thermogenesis [32–34]. Recent studies on humans have demonstrated an inverse correlation between the amount of brown adipose tissue and BMI, hence suggesting a potential role of brown adipose tissue in adult human metabolism [35–37]. Seasonal variations in the prevalence of active brown adipose tissue or the thermogenic potential of subcutaneous white adipose tissue has also been demonstrated. These observations strengthen the hypothesis that these organs act as a critical mediator of ambient temperature and obesity [38, 39]. Recently, Bakker et al. reported that south Asian populations exhibited a lower resting energy expenditure, non-shivering thermogenesis and brown adipose tissue volume compared to Caucasians in a prospective case-controlled observation study and suggested that these findings might underlie higher susceptibility to metabolic disturbance in Asian populations [40]. Thus the relative contribution of brown adipose tissue in the association between ambient temperature and obesity prevalence must be further elucidated in the future.

There are several limitations to our study. As this was a cross-sectional study, we were unable to determine the cause-effect relationship between ambient temperature and the prevalence of obesity. In addition, because our database only contained the subject’s current area of residency, people who had moved to or from different areas could not be excluded. Therefore, we could not rule out a potential reverse causation between obesity and ambient temperature of residential regions. Moreover, as our study was conducted only among Korean subjects, further studies in different countries and among different ethnic groups as well as a wider range of temperatures are needed to confirm our data. Subjects in the highest quintile of the MAT group had a significantly higher age and lower income level compared to those in the lower four quintiles of the MAT group. These two factors alone might have influenced the prevalence of obesity. However, the highest quintile of the MAT group demonstrated independent odds for obesity or abdominal obesity after statistical adjustment for age and income level. Moreover, different latitudes of observational areas might have influenced the prevalence of obesity, considering Bergmann’s rule which states populations of larger size are found in colder environments and those of smaller size are found in warmer regions. However, Foster et al. [41] suggested that Bergmann’s rule might hold true only when there are large latitudinal (> 50°) and temperature (> 30°C) difference among groups. In our study, the range of latitude was narrow (33–38°), suggesting little effect of latitude as confounding variable. Lastly, influence of ambient temperature on appetite and energy intake might lead to altered energy homeostasis [42]. However we were unable to account for energy intake in this study. The strengths of our study include the use of large-scale nationally representative data about Korea from the NHIS and meteorological data obtained during the previous 30 years. Other than one study of the Tibetan population [12], which focused on the effect of altitude on obesity prevalence and suggested a possible association with temperature, our study is the first to investigate the Asian population and evaluate the association between exposure to daily life temperature and obesity.

In conclusion, this study shows a significant association between ambient temperature and prevalence of obesity among the Korean population when controlled for several confounding factors. Cold-induced thermogenesis may be a possible explanation for this phenomenon and should therefore be the subject of further study.
